# Assessing Rhythmic Visual Entrainment and Reinstatement of Brain Oscillations to Modulate Memory Performance

**DOI:** 10.3389/fnbeh.2020.00118

**Published:** 2020-07-16

**Authors:** Michel J. Wälti, Daniel G. Woolley, Nicole Wenderoth

**Affiliations:** ^1^Neural Control of Movement Lab, Department of Health Sciences and Technology, ETH Zürich, Zurich, Switzerland; ^2^Cognition, Perception and Behaviour in Urban Environments, Future Cities Laboratory, Singapore-ETH Centre, Singapore, Singapore; ^3^Neuroscience Center Zurich (ZNZ), University and ETH Zürich, Zurich, Switzerland

**Keywords:** memory, neural reinstatement, steady-state evoked potentials, entrainment, neural oscillations

## Abstract

The human brain’s ability to store information and remember past events is thought to be orchestrated by the synchronization of neuronal oscillations in various frequency bands. A vast amount of research has found that neural oscillations in the theta (∼4–7 Hz) and alpha (∼8–12 Hz) bands play an important role in memory formation. More specifically, it has been suggested that memory performance benefits if the same oscillatory pattern is present during encoding and retrieval. However, the causal relevance of these oscillations is not well understood. Rhythmic sensory stimulation is thought to entrain ongoing brain oscillations and modulate associated functions (e.g., memory formation). In the present study, we used rhythmic visual stimulation at 6 and 10 Hz to experimentally modulate the memory encoding process in a recognition memory task. In addition, we reinstated oscillatory activity from the encoding episode during retrieval, which has been hypothesized to result in memory performance improvements compared to non-reinstated conditions and incongruent reinstatement. Contrary to our hypothesis, we find no effect of neural entrainment during encoding on subsequent memory performance. Likewise, memory retrieval does not benefit from neural reinstatement. The results are discussed with respect to methodological challenges of rhythmic sensory stimulation as a means to alter cognitive processes and induce context-dependent memory effects.

## Introduction

Memory processes in humans are characterized by the ability to store and mentally reconstruct episodic information. A vast amount of research has found brain activity to be an important mechanism for the formation and reactivation of memories, especially neural oscillations (for reviews, see Klimesch, 1999; Düzel et al., 2010; Fell and Axmacher, 2011).

The formation of episodic memories has been associated with neuronal oscillatory activity in the theta (∼4–7 Hz), alpha (∼8–12 Hz), beta (∼15–30 Hz), and gamma (>30 Hz) frequency bands (Friese et al., 2013; Hanslmayr and Staudigl, 2014; Köster et al., 2018). The encoding of visual information has been specifically linked to increases in theta and gamma power and decreases in alpha power (Klimesch et al., 1997; Clarke et al., 2018). Theta is known to be enhanced during successful encoding in context-dependent memory tasks (Addante et al., 2011; Staudigl and Hanslmayr, 2013; Clarke et al., 2018) because it is believed to facilitate associative binding of perceptual information (Hanslmayr et al., 2011; Clouter et al., 2017). Moreover, it has been argued that memory benefits not from enhancing theta amplitude *per se* but rather from the associated strengthening of theta–gamma phase-amplitude coupling in the human cortex (Canolty et al., 2006; Friese et al., 2013; Köster et al., 2014, 2019), which is thought to be responsible for ordering and integrating novel perceptual information into existing memory networks (Buzsáki, 2006). Reduced alpha power is thought to reflect cognitive and attentional gating processes during encoding by disinhibiting relevant brain areas (Klimesch et al., 1997; Jensen and Mazaheri, 2010; Köster et al., 2019).

Successful remembering not only is determined by neuronal processes during encoding but also depends on how the information is later retrieved. This claim originates from early studies showing that memory performance depends on the overlap of the encoding and retrieval situation (Tulving and Thomson, 1973; Godden and Baddeley, 1975; Morris et al., 1977). This so-called encoding specificity principle states that memory performance is higher if contextual features during retrieval match those of the encoding episode compared to non-matching situations (for a review, see Smith and Vela, 2001). Intriguingly, neural activity has been shown to reveal similar patterns during memory tasks. This neural reinstatement hypothesis claims that patterns of cortical activity during encoding are reinstated during successful retrieval (Nyberg et al., 2000; Wheeler et al., 2000). Recent studies using electrophysiological measures of brain activity [electroencephalography (EEG) or magnetoencephalography (MEG)] found evidence for neural reinstatement by demonstrating the reoccurrence of oscillatory patterns present at encoding during retrieval (Jafarpour et al., 2014; Staudigl et al., 2015; Waldhauser et al., 2016). Wimber et al. (2012) reported that reinstated neural patterns are not restricted to naturally ongoing brain activity but can also represent previously evoked oscillations (Wimber et al., 2012). During encoding, participants were exposed to visual flickering (6 or 10 Hz), which is known to produce steady-state visually evoked potentials (SSVEPs) in visual areas of the brain at the same frequency as the visual input (Regan, 1977). Intriguingly, during the subsequent recognition task which was performed with a neutral background, the authors found that successful retrieval was accompanied by intrinsically evoked neural activity at the same oscillatory frequency as extrinsically evoked during encoding (Wimber et al., 2012). This finding confirms the view that neural reinstatement supports memory recall and provides further evidence for the possibility of tagging memories with rhythmical sensory stimulation.

Although Wimber et al. (2012) found no effect of visual stimulation on memory performance, others revealed a correlational relationship between neural oscillations and memory processes, with modulated memory performance resulting from experimentally altered neuronal activity. Transcranial alternating current stimulation (tACS) is a common method to alter neuronal activity and has been reported to affect the power, phase, and frequency of ongoing brain oscillations (e.g., Zaehle et al., 2010; Cecere et al., 2015). While early research used tACS to target consolidation processes during sleep (Marshall et al., 2006), more recent findings aimed to alter neural activity during encoding and retrieval (Javadi et al., 2017; Nomura et al., 2019). Applying tACS during a declarative memory task at gamma frequencies (60 and 90 Hz) to the left dorsolateral prefrontal cortex, Javadi et al. (2017) revealed that applying the same stimulation frequencies during encoding and recognition caused a significant memory improvement relative to sham stimulation (Javadi et al., 2017).

An alternative method to entrain brain oscillations is rhythmic sensory stimulation as described above. Compared to tACS, this method has the advantage that no electrical artifacts are induced, thus EEG recording during stimulation is feasible (for a review, see Vialatte et al., 2010). Regarding processing of visual stimuli, research using an object-recognition task has shown that SSVEPs in the theta band are increased for unfamiliar stimuli, while SSVEPs in the alpha band revealed a similar increase for familiar stimuli (Kaspar et al., 2010). Applying audiovisual entrainment, a recent study found that in-phase visual and auditory theta stimulation enhanced associative memory formation (Clouter et al., 2017). This finding provides causal evidence that memory formation relies on the synchronization of sensory inputs in the theta band. A similar study was conducted by Roberts et al. (2018), who exposed participants to audiovisual theta entrainment during the consolidation phase of an episodic memory task. Confirming the crucial role of theta oscillations in memory formation, they found that entrainment resulted in increased theta power during memory retrieval, which was associated with better memory performance (Roberts et al., 2018). In order to modulate memory performance, Köster et al. (2019) had visual objects in an object-recognition task flicker at individually adjusted theta and alpha frequencies. In line with the hypothesized effects of the chosen frequency bands, they found that theta enhanced the formation of novel memories, in contrast to alpha stimulation. Their EEG results revealed that this behavioral effect could not be explained by changes in theta power alone but by theta–gamma phase-amplitude coupling in widespread cortical networks (Köster et al., 2019). Regarding the memory effects of theta and alpha entrainment, it has to be noted that encoding without visual stimulation resulted in the best overall memory performance, suggesting that flickering had primarily a disruptive effect, confounding the potential effect of neural entrainment on memory formation.

Taken together, neural oscillations during encoding of new information and the match between oscillatory patterns during encoding and subsequent retrieval play an important role for episodic memory formation and performance. Additionally, rhythmic sensory stimulation is thought to entrain ongoing brain oscillations and modulate associated functions (e.g., memory formation). Building on the abovementioned findings, in the present study, we used rhythmic visual stimulation in the theta (6 Hz) and alpha (10 Hz) frequency bands during encoding to experimentally modulate the memory formation process in a recognition memory task. In addition, we hypothesized that reinstating the same oscillatory activity during encoding and retrieval *via* sensory stimulation would result in memory performance improvements compared to non-reinstated conditions and incongruent frequency reinstatement.

## Materials and Methods

### Participants

A total of 34 healthy participants with normal or corrected-to-normal vision were recruited and participated in the experiment. One participant was excluded from the analysis due to technical problems. Three others did not reach the minimum required performance rate of 80% correct responses during the encoding task and were excluded because insufficient attention was suspected. This resulted in a final sample of 30 participants (female: 17; age: M ± SD = 23.8 ± 4.5 years). The study protocol was approved by the local ethics committee of the canton of Zurich and was conducted in accordance with the Declaration of Helsinki. Participants provided informed consent and were briefed on the study procedures. Participants were naive with regard to the hypothesis of the study.

### Design and Procedure

Participants were comfortably seated in a dark sound-attenuated room approximately 100 cm away from a (27-inch) computer screen. The experimental procedure was adapted from a previous study (Wimber et al., 2012) and consisted of two consecutive parts: encoding and retrieval of words presented on a computer screen. For this, 360 German nouns were derived from the Berlin Affective Word List (BAWL-R). Selected words were restricted to consist of one to three syllables, an imageability rating of at least 5.0, and an arousal rating of less than 3.0. Assignment of words to experimental conditions was randomized for each participant.

#### Encoding

**FIGURE 1:**
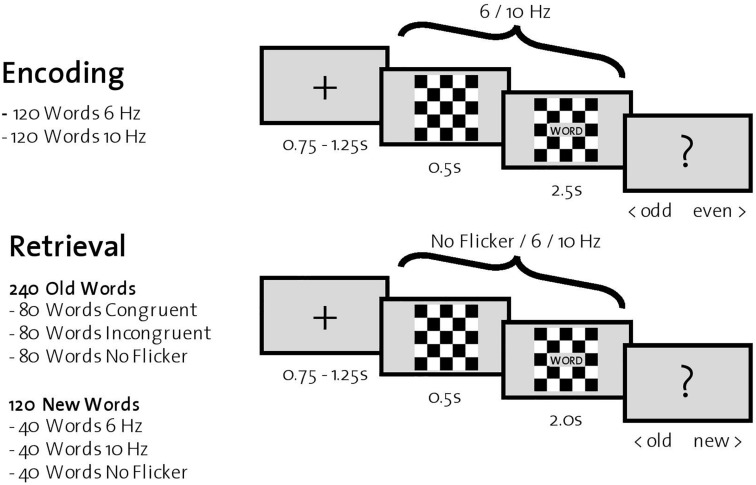
Experimental procedure. **Top:** One trial during encoding. Participants were instructed to indicate whether the presented word had an odd or even number of syllables. **Bottom:** One trial during retrieval, where all previously encoded words (old words) and 120 new words were presented successively. For each word, participants were instructed to indicate whether it was old or new.

During encoding, 240 words were successively presented in the center of a computer screen with a gray background. Each trial started with the presentation of a fixation cross (0.75–1.25 s), followed by a black-and-white checkerboard (Figure 1). In half of the trials (120 words), the checkerboard flickered at 6 Hz; in the other half, at 10 Hz. The order of the trials was randomized. After 0.5 s, a word appeared in a gray box in front of the flickering checkerboard for 2.5 s. Participants were instructed to count the number of syllables for each word and indicate *via* a button press during the subsequently presented screen (depicting a question mark for 1 s) whether the number of syllables was odd or even. After the encoding phase of all 240 words, participants were instructed to execute a 5-min distraction task (counting backward) before the retrieval phase started.

#### Retrieval

Retrieval trials consisted of the previously presented 240 words (old words) together with the remaining 120 words that participants had not yet seen (new words). Each trial started with a fixation cross in the center of a gray screen (0.75–1.25 s), followed by a black-and-white checkerboard. After 0.5 s, a word appeared in front of the checkerboard for 2.0 s. The checkerboard flickered in one third of trials consisting of old words (=80 words) at the same frequency as during encoding (congruent trials), in another third at the other frequency (incongruent trials), and finally in the last third of trials without flickering (no flicker trials). In trials consisting of new words, one third (=40 words) was flickering at 6 Hz, another third at 10 Hz, and the last third without flicker. Again, the order of the trials was randomized. After word presentation, a question mark appeared in the center of the screen for 1.5 s, and participants were instructed to indicate with a button press whether the word was old (one that was previously encoded) or new.

### Data Analysis and Statistics

Our behavioral outcome measure compared hit rates across different encoding and retrieval conditions (see “Results”). For each condition, hit rates were calculated as the number of correctly remembered old words divided by the total number of presented old words during retrieval. A repeated-measures ANOVA in SPSS Version 25 (IBM, United States) tested for statistical differences in memory performance between entrainment conditions. In addition, Bayes factors (BF_10_ and BF_01_) were derived using JASP software Version 0.8.6^1^. The main advantage of Bayes statistics over a frequentist approach is the expression of evidence in favor of the null (H0) or alternative hypothesis (H1) (Van De Schoot et al., 2017).

## Results

### No Effect of Encoding Frequency on Encoding Performance

To rule out differences in recognition based on attentional processes during the encoding phase of the experiment, we first analyzed the encoding performance across both entrainment conditions (6 and 10 Hz). Encoding performance was determined as the rate of correct responses in the syllable counting task. Although on average, accuracy during the 10-Hz flicker was higher (*M* = 0.95, SD = 0.04) compared to that of the 6-Hz flicker (*M* = 0.94, SD = 0.04), a paired-sample *t*-test revealed no statistical difference between the two entrainment frequencies during encoding on syllable counting accuracy [*t*(29) = −1.366, *p* = 0.182]. This result suggests comparable levels of attention during both entrainment conditions.

### No Effect of Encoding Frequency on Retrieval Performance

**FIGURE 2:**
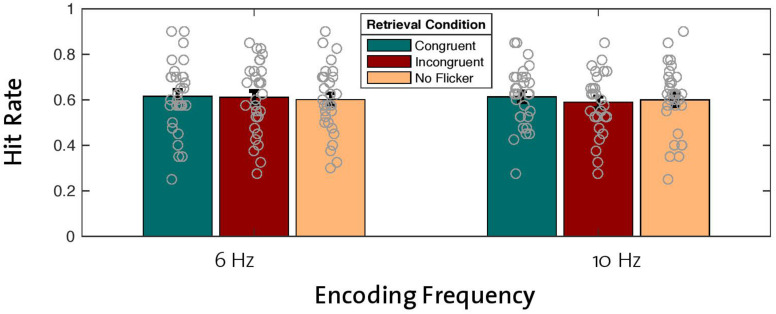
Effects of encoding frequency and retrieval conditions on hit rates for old words. A 2 × 3 repeated-measures ANOVA revealed no main effect of encoding frequency or retrieval condition on hit rates. Gray circles represent individual data points [error bars = standard error of mean (SEM)].

With the aim of replicating the previously reported effect of enhanced memory performance after theta entrainment during encoding compared to alpha entrainment (Köster et al., 2019), we analyzed hit rates across old words that were encoded with the 6-Hz flicker and compared them with hit rates for old words that were encoded with the 10-Hz flicker. Note that this analysis included old words regardless of retrieval condition (congruent, incongruent, no flicker). A repeated-measures ANOVA (2 × 3 factors: two encoding frequencies with three retrieval conditions) revealed no main effect of encoding frequency on hit rate [*F*(1,29) = 0.563, *p* = 0.459; Figure 2], thus failing to replicate the beneficial effect of theta compared to alpha entrainment. Bayes statistics revealed that the null effect of the encoding frequency was 4.9 times more likely compared to the alternative hypothesis (BF_10_ = 0.209, BF_01_ = 4.918), thus suggesting moderate evidence for the null hypothesis (H0) (see Jeffreys, 1961).

### No Effect of Neural Reinstatement on Memory Performance

To test the hypothesized effect of reinstated neural oscillations on memory performance, we looked at the main effect of retrieval condition in the same ANOVA described in the previous section (2 × 3 factor repeated-measures ANOVA). Contrary to our hypothesis, differences in old word hit rates between retrieval conditions (congruent, incongruent, no flicker) across both entrainment frequencies revealed that reinstatement of neural oscillations did not have a beneficial effect on hit rates [*F*(2,28) = 0.513, *p* = 0.604; Figure 2]. Bayes factors confirmed this finding by revealing moderate evidence in favor of the null hypothesis (BF_10_ = 0.109, BF_01_ = 9.154). In addition, our analysis revealed no significant interaction effect between encoding frequency and retrieval condition [*F*(2,28) = 0.398, *p* = 0.676; BF_10_ = 0.127, BF_01_ = 7.862].

## Discussion

Previous research has revealed that (i) theta and alpha neural oscillations play an important role in human memory formation, and (ii) reinstating the same oscillatory patterns during retrieval as during encoding improves remembering of stored information (for a review, see Hanslmayr and Staudigl, 2014). In addition, a recent study found rhythmic sensory stimulation during encoding to modulate relevant brain oscillations, resulting in altered memory performance (Köster et al., 2019). The aim of the present study was to build on these previous findings and to expand the suggested relevance of rhythmic sensory stimulation as a tool for neural entrainment by revealing the effects of neural reinstatement on memory performance. We used rhythmic visual stimulation in the theta (6 Hz) and alpha (10 Hz) frequency bands during recognition memory task encoding and induced reinstatement of oscillatory activity during retrieval with matching (congruent) or mismatching (incongruent) frequencies. In contrast to our hypotheses, we found no effect of entrainment on memory performance during encoding and no influence of matching entrainment frequencies during encoding and retrieval.

### Mixed Findings Regarding Visual Entrainment on Memory Performance

Influencing memory performance by entraining neural oscillations *via* rhythmic sensory stimulation has been a long-standing idea. For the visual system, Köster et al. (2019) reported recently that theta entrainment during encoding enhances subsequent memory retrieval compared to alpha entrainment. However, two other studies following a similar rationale could not reproduce the effect reported by Köster et al. (2019). First, Wimber et al. (2012) entrained visual theta (6 Hz) or alpha (10 Hz) during encoding but found no difference in recognition performance between words that were encoded at 6 Hz and words that were encoded at 10 Hz. The authors do not report whether such an effect was initially hypothesized, however, the choice of the two stimulation frequencies from well-known memory frequency bands (theta and alpha), which have opposite effects on memory performance, suggests that, at least partly, a behavioral effect was expected. To our knowledge, only one other study used a similar approach of rhythmic visual entrainment in a recognition memory task. Lewis et al. (2018) used 6- and 15-Hz visual entrainment during word encoding and also found no effect of stimulation frequency on memory performance. The findings of these latter studies are in line with our results which also failed to reveal a significant effect of 6- vs. 10-Hz visual stimulation during encoding. Two main methodological differences between the study from Köster et al. (2019) and the others (including our study) need to be pointed out.

First, all studies included theta and alpha (or low beta, see Lewis et al., 2018) stimulation frequencies; however, Köster et al. (2019) used individually adjusted stimulation frequencies from theta (3–8 Hz) and alpha (8–13 Hz) ranges. Previous research has shown that neural entrainment is partly dependent on the match of the frequency of the stimulating input and the frequency of endogenous neural oscillations (Notbohm et al., 2016; Gulbinaite et al., 2017; Wälti et al., 2019a). Potentially, our 6- and 10-Hz stimulation rhythms did not have the anticipated entrainment effect on neural oscillations in participants with slightly divergent individual theta and alpha frequencies. Moreover, it has been shown that improved memory performance does not primarily result from reinstating theta and/or gamma band power *per se* (Köster et al., 2014) but rather from increasing phase-amplitude coupling between the prefrontal theta phase and the posterior gamma amplitude (see Friese et al., 2013; Köster et al., 2014). Future studies using this paradigm would likely benefit from electrophysiological measurements (e.g., EEG) to target individual peak frequencies in the entrained oscillation bands and from additional analyses testing whether neural entrainment effectively modulated theta–gamma coupling which appears to be a key mechanism in memory formation and retrieval.

The second and possibly crucial difference to Köster et al. (2019) was the to-be-learned stimuli. While the present study, Wimber et al. (2012), and Lewis et al. (2018) used words, Köster et al. (2019) used colored object pictures (e.g., animals, tools). Early research found that recognition for pictures was enhanced compared to words (e.g., Paivio et al., 1968; Snodgrass et al., 1972) and elicited different brain regions (Köster et al., 2018) arguably because pictures automatically engage stronger associations with previously stored memories, thus enabling more elaborate encoding (Grady et al., 1998). In a positron emission tomography (PET) study, Grady et al. (1998) compared the neural correlates of encoding words vs. pictures. While picture encoding revealed greater activity in the medial temporal cortex, which is known to be a crucial brain region for episodic memory formation, words activated regions in the left hemisphere, known for their involvement in language tasks (Grady et al., 1998). Although differences in the neural processes between encoding words and pictures have been established, how these findings explain the mixed results in our study and Köster et al. (2019) remains elusive. On one hand, neural entrainment of pictures possibly induces rhythmic activation not only in primary visual cortex but spreads to higher level processing areas (e.g., the medial temporal lobes which are involved in memory formation). On the other hand, flickering during encoding of words might reveal no spread of activation or only to areas of linguistic processing, thus not modulating memory processes. More research, ideally using invasive electrophysiological measures [e.g., electrocorticography (ECoG)], would be needed to evaluate the rhythmic spreading of steady-state evoked potentials. Köster et al. (2018) found in a recent study that theta–gamma phase-amplitude coupling was observed in memory tasks using picture–color associations, but not when words were used as stimuli, further highlighting the importance of the encoding stimuli (Köster et al., 2018). However, an increase in theta oscillations, as well as a suppression of the alpha rhythm, was found regardless of encoding condition, which would suggest that our null results cannot be solely explained by the use of words as encoding stimuli.

Besides the main differences in the methodologies between the work reported here and Köster et al. (2019), other factors which distinguish the two studies might have led to the mixed results. First, in the present study, participants were asked to count syllables during the encoding of the presented words (adapted from Wimber et al., 2012). In comparison to Köster et al. (2019), which used a dot detection task, this procedure arguably was cognitively more demanding and might have overshadowed the effect of the entrainment by stronger top-down processes (e.g., suppression of alpha oscillations). Second, Köster et al. (2019) flickered the to-be-learned objects, while we used a flickering checkerboard in the background of the words. Again, this was adapted from Wimber et al. (2012), who provided electrophysiological evidence that this procedure evoked robust SSVEPs.

In addition to methodological differences to previous reports, we acknowledge further limitations in our study. Although our study design was adapted from previous research, we recognize that the high number of experimental conditions led to a reduced number of trials per condition (240/6 = 40). Further, more insight into the quality of the memory processes could have been achieved by using a remember–know study design, which allows participants to state different degrees of (un-)certainty when remembering a stimulus.

### Mixed Findings Regarding Neural Reinstatement and Visual Flicker as Contextual Feature During Memory Formation

The fact that contextual features surrounding the encoding event reemerge during later retrieval implies a process at the encoding stage which serves to bind items to their contextual features. This has been suggested to be a key component to form episodic memories (Tulving, 1972; Tulving and Thomson, 1973). Visual flickering, used in the present study, not only entrains brain oscillations in visual areas but also represents a visual context that arguably would enhance memory performance when later reinstated (for a review on environmental context-dependent memory, see Smith and Vela, 2001). This idea stems from previous research showing that even simple visual context modulations (e.g., background color of a computer screen) can produce a context-dependent memory effect in humans (Murnane and Phelps, 1993; Isarida and Isarida, 2007). It is also in line with experimental evidence for reactivated neural activity from the encoding period during successful retrieval (for a review, see Hanslmayr and Staudigl, 2014). Causal evidence supporting this idea was provided by Javadi et al. (2017) who externally induced oscillatory rhythms (with tACS) and showed that this neural reinstatement modulated memory performance (Javadi et al., 2017). However, a closer exploration of their results suggests that the comparison between active and sham stimulation conditions, which revealed an effect on memory performance for congruent but not for incongruent simulation, was, at least partly, driven by high variability in the sham conditions. In a similar vein, Wimber et al. (2012) claimed that successful retrieval is associated with reactivating the oscillatory activity of the encoding stage (Wimber et al., 2012). However, a later study from Lewis et al. (2018) was not able to replicate the findings of reinstated SSVEPs during successful retrieval most likely because Wimber et al. (2012) were too lenient in applying statistical correction for multiple comparisons. They conclude that reinstatement effects of SSVEPs are not robust enough to be used as a reliable index of lexical activation during language processing (Lewis et al., 2018).

### Is Rhythmic Sensory Stimulation a Feasible Method to Modulate Memory Processes?

A vast amount of research has provided evidence for the involvement of brain oscillations in human memory processes. However, the somewhat naive assumption that single frequency bands in separated brain regions can be causally associated with memory processes has been challenged in recent years. In their review, Hanslmayr and Staudigl (2014) report that there is evidence for the involvement of each frequency band (from 3 to 100 Hz) with correlations for both increasing and decreasing amplitudes in memory formation (see also Düzel et al., 2010). The authors conclude that brain oscillations during encoding primarily represent cognitive and perceptual processes, i.e., a mix of bottom-up and top-down governed effects. Further, they argue that successful memory retrieval is related mainly to two classical concepts of episodic memory (Hanslmayr and Staudigl, 2014). First, the level of processing during encoding (Craik and Lockhart, 1972), and second, the encoding specificity principle, which states that information is more likely to be retrieved if conditions at the time of retrieval are similar to those at the time of encoding (Tulving and Thomson, 1973). In our study, participants were engaged in a syllable-counting task during encoding which led to incidental learning of the presented words and arguably an equivalent level of processing across conditions (6 or 10 Hz encoding). Confirming this assumption, no difference was found between the two encoding conditions in the accuracy in the syllable-counting task. Nonetheless, we found no difference on memory performance between theta and alpha entrainment. A possible reason for this lack of an effect could be an oversimplified understanding of the involvement of brain oscillations in the formation of memories. While most early studies focused on specific frequency bands (e.g., theta, gamma, alpha) and single measurement parameters (e.g., power, coherence), recent studies have revealed more complex mechanisms (e.g., cross-frequency phase-amplitude coupling) (see Düzel et al., 2010; Hanslmayr and Staudigl, 2014). These heterogeneous relationships between oscillatory dynamics and various forms of memory formation make modulations with external rhythmic stimulation extremely difficult.

While reinstatement of visual flickering in our studies provides alterations in similarity between the encoding and retrieval situations, such narrow-band neural oscillations in a single sensory system might not be sufficient to reveal the hypothesized effects of encoding specificity. In addition, we found recently that even fully surrounding environments and on-screen presentations of colors or landscape pictures were consistently unable to evoke context-dependent effects on memory (Wälti et al., 2019b). Taken together, these findings suggest that modulation of visual contexts alone is not sufficient to evoke effects on the formation and retrieval of memories. The process of memorizing information always consists of a variety of sensory, cognitive, and emotional features (Fernandez and Glenberg, 1985). Sensory stimulation would have to be presented to various sensory systems simultaneously, but even then, ongoing cognitive (top-down) processes, as for example individual mnemonic strategies, might overshadow possible effects.

## Conclusion

In our study, memory performance was not modulated by visually induced neural entrainment of theta (6 Hz) and alpha (10 Hz) frequencies, and we observed no effect of reinstated entrainment frequencies during retrieval. The heterogeneity of brain oscillations and regions involved in these complex processes makes targeted entrainment *via* rhythmic sensory stimulation difficult, and further cognitive top-down processes during encoding and retrieval are most likely expected to overshadow entrainment effects. However, more sophisticated approaches of sensory entrainment might reveal more promising results. The use of individually adjusted stimulation signals (e.g., in a closed-loop setup) applied to multiple sensory systems, possibly with cross-frequency coupling, could provide stronger entrainment effects.

## Data Availability Statement

The datasets generated for this study are available on request to the corresponding authors.

## Ethics Statement

The studies involving human participants were reviewed and approved by the Kantonale Ethikkommission Zürich. The patients/participants provided their written informed consent to participate in this study.

## Author Contributions

MW designed and performed the research and analyzed the data. MW, DW, and NW wrote the manuscript. All authors contributed to the article and approved the submitted version.

## Conflict of Interest

The authors declare that the research was conducted in the absence of any commercial or financial relationships that could be construed as a potential conflict of interest.

## Footnotes

1 https://jasp-stats.org/
